# Pen Pictures of Classic Men and Classic Scenes in Europe

**Published:** 1871-03

**Authors:** 


Pen Pictures of Classic Men and Classic Scenes in Europe.—We take the following extracts from an interesting article by Joseph Pancoast, M.D., in the *Richmond and Louisville Journal,* January, 1871:

"There are, as you are aware, but four great medical and surgical schools in the world—the French, German, British and American. In Spain, which I thoroughly visited, I met, it is true, with many accomplished gentlemen in our profession, who displayed, in my intercourse with them, the characteristic courtesy of that once great people. But in that country every scientific effort toward advancement in our profession is so entrammeled by the deplorable ignorance and bigotry which exist even among the rulers of society, that the Spanish school has not been able to attain any elevated rank, and is made up mainly of French doctrines at second hand.

The Italian school is immeasurably in advance of the Spanish, though sunk below the position it once occupied, when, in the days of Fabricius, Malpighi, Morgagni, and Scarpa, it was the anatomical and surgical teacher of the world. Not that Italy is now altogether devoid of prominent medical men; I have found able surgeons in Naples, Florence, and Bologna, and you are all familiar with the reputation of Pacchini, of Florence, the discoverer of those singular nervous bodies that bear his name, whom I found one of the most intelligent, warm hearted and interesting men that I ever had the happiness to meet.

As I walked through the ancient palatial seats of learning at Bologna and Padua, I could but mourn over their decline in reputation from those bright and palmy days of grandeur, when their halls were thronged with the most aspiring students from all parts of Europe. The escutcheons belonging to the name and race of the more distinguished still hang in great numbers upon the walls, among which I observed that of the celebrated Harvey, the discoverer of the circulation. He was a student of Padua, and gleaned from the teachings of Fabricius, of Aquapendente, an insight into the mysteries of the vascular system, which led to his discovery of the circulation, and made his name famous for all time.

This lecture-room of old Fabricius, the authorities have had the good taste to preserve unchanged down to the present time. What appearance, think you, it presents?—a spacious theatre, with cushioned seats, on which his audience could recline with ease? No, indeed. It is but a tall, narrow, circular chamber, much smaller than the old lecture-room of the Pennsylvania Hospital, like a punch-bowl in shape, with narrow galleries running around, in which the students could merely squeeze themselves in a standing posture, in order to observe the demonstrations of the great man below.

But there is hope yet for Italy. Her skies remain as fair, and her soil as fertile as ever, and a brighter sun shines in her political horizon. There is yet hope for the Italian people, for they still honor their great names of the past. So I felt as I stood in the beautiful lecture-room of Bologna."

A visit to the clinic of Prof. Hebra, in Vienna, is thus described:

"We will go now with the small crowd that is hurrying to attend the world-renowned clinic of Prof. Hebra, the man who justly holds the highest place in the world's estimation for his skill in the diagnosis and treatment of the complicated and multifarious diseases of the skin, who has, in his career, treated more than 10,000 cases of smallpox and other cutaneous diseases in proportion, and given to the world pictorial representations of them that have never been equaled.

Punctual to the hour affixed for his private course, in rolls a short man, with a round and portly person, and features expressive of great sprightliness and intelligence; this is Prof. Hebra. He hangs up his hat on a peg, a fashion in which all follow him. His rostrum is a chair placed within on a slightly raised platform about twelve feet square. Upon this platform the patients are introduced, one by one, and passed along the inner side of the railing so as to exhibit their diseases to the students who are ranged round it without.

Most of the varieties of skin disease pass before him in the course of these lectures—syphilis, psoriasis, itch, lupus, prurigo, *et id genus omne*, are diagnosed with wonderful acumen, and judiciously treated. Your trust in his accuracy cannot be withheld. For in cases where the nature of the disease is ambiguous or mixed, and they are often so, he has the manliness to tell you that he must withhold his opinion for another day, or until he sees the proper grounds for a diagnosis.

The examination of each case is, however, thoroughly made. No vestments stand in the way, whatever the sex; there is at least no mock sentimentality on either side. Occasionally a man may enter 6 feet high, perhaps, step up upon the platform with military precision, and, dropping a loose gown from about him, make a formal profound obeisance in a state as naked as was Adam before he began tailoring with fig leaves.

Prof. Hebra believes in the unity of syphilis, as does his colleague Prof. Sigmund, and uses mercury and sulphur in its treatment as we do. But the mode of using mercury, especially in the secondary and tertiary forms, at present in vogue with both these gentlemen, is to inject under the skin, once daily, the tenth part of a grain of corrosive sublimate for an adult, and the thirtieth for a child. Small as this amount is, it does not do to exceed it, or it will excite nausea and vomiting. It is thrown in, in solution, with the hypodermic syringe, under the skin of the buttocks, at the lower point of the scapula, or over the insertion of the deltoid muscle. They will tell you, however, that the value of this process is yet under trial.

The plan is to continue the treatment for twenty seperate injections, and then observe the results; if necessary, recommencing the injections again after a few days. Small as is the amount of sublimate thus introduced, it can after ten days be detected in the urine. And this is not strange; for the use of chemical tests is now brought to such a great degree of perfection that it is boldly asserted that the presence of a .020 part of a grain of quinia can be detected in the urine by the means of the spectrum and the passing of an electric current through the fluid, catching the quinia on a piece of gold leaf.

The well-known compound sulphur bath of Fleming, the Surgeon-in-Chief to the Belgian armies, is the one commonly used, especially in tertiary syphilis and psoriasis. It is applied, according to the case, from two to six hours, and if you look around, you will see at any time, almost, a dozen male patients floating around like so many seals in one common bath-tub. It is his treatment, too, for the itch, which he says he kills in half an hour, killing at once the insects and addling the strings of eggs which they lay along so nicely in little galleries beneath the skin. It says little for the cleanliness of the habits of the people of Vienna that this disgusting disease should be so common there. It is so often found, even among the higher classes, that Hebra will tell you he finds it convenient to use a popular preparation, charged with aromatics, that he calls his aristocratic itch ointment. Even the cats and dogs of Vienna, he says, suffer with the same disease, produced by the same insect. In bad cases of pemphigus, when the bullæ have given place to a delicate and imperfect cuticle, he lays his patients in a bath of perpetually flowing water, and when taken out, they are dressed in an India-rubber vest and pantaloons, and even the face covered with pieces of India-rubber cloth. Bad cases of hopeless burns are put into this perpetual bath, where they find themselves easier than elsewhere, and, as Hebra will tell you, die very comfortably. But it would be tedious to go into a description of this bath, with its inclined bed and its windlass arrangement, or of his famous tar bath, or of his habit of scrubbing the bodies of his patients over with the strongest kind of soap when the skin is insensitive and incapable of absorption. When there is much cutaneous sensitiveness, then the cocoa-nut oil soap is used. A friend in Vienna told me he was using, with excellent effect, this coconussenoilsodaseife in the case of a Constantinopolitanisherdudelsackpfeifer—that is to say, he was using the cocoa-nut oil soda soap in the case of a Constantinople bagpipe player.

Some simple rules Hebra follows closely in getting at the differential diagnosis between syphilis and common psoriasis. He tells you that syphilitic eruptions never itch like psoriasis; that they come frequently in the bend of the arm and in the ham, psoriasis never; and that syphilitic sores always produce a loss of tissue, and that others usually do not.

Prof. Sigmund exonerates our country from the supposed opprobrium of having originated the syphilitic poison. He says it is described in the Bible, as many had previously supposed, and showed me a pamphlet just published by a learned Jewish physician, in which it is said that Luther, out of commendable modesty, has purposely mistranslated the passage.

To Prof. Sigmund we are indebted for the knowledge of the important fact that the raw ulcerations on the side of the tongue, and called syphilitic psoriasis, are very contagious. From ignorance on this point, he has seen the most deplorable consequences follow, as many of us have done—the infected child poisoning the nurse at whose breast it sucks; the diseased nurse poisoning the child, by first chewing its food in her own mouth, and the most innocent and pure-minded have their systems infected simply by the kiss of a brother or other near relative.

But let us now shake off any infection that may attach to our garments in this place, and move on in another direction."

"We pass, on our return, near the obstetrical department, under the care of Profs. Shote and Braun.

We will enter there. It contains 600 beds, and 10,000 births take place there yearly. The patients are taken in only during the last month of their pregnancy. Graduates who pay the private fee, and bribe the nurses, get cases in abundance, even those requiring the use of the forceps, and are able to secure patients in advance, by writing their names over the beds. Twenty placentas are lying on the table, the product of the preceding night.

The patients are carried into one room to be confined, and are transported back on a litter to another when the trouble is over.

No belly-bandage is put on ordinarily. If there is after-hæmorrhage, cold is applied to the abdomen, or cold water injections made into the vagina; if it prove very obstinate, then one or two drachms of the tincture of the sesquichloride of iron, diluted in a pint of water, is thrown into the uterus. If bleeding is still continued, then a belly-bandage is applied, a proceeding which I believe few judicious obstetricians in this country would so long delay. Every infant is swathed like a mummy, or an Indian pappoose, and so kept for months, bound up so tightly in bandages that they rock it by rolling it on the bed as they would rock a cradle. The attendants even seemed to have no idea that infants were differently treated in other parts of the world. There are no regular lectures here; nothing purely didactic in midwifery or any other branch. What the chance practice offers, they lecture on, either at the bedside, or by adjournment to the lecture-room adjoining, so that if during the course there were five cases of placenta prævia, there would be five lectures on it If no case, no lectures on the subject. This is one of the great faults of the institution.

There is a great chattering now in the lecture-room, as if a big school had broken loose.

Look in there. You will see sixty or seventy women, all students of this branch, between the ages of 20 and 35; some sitting, some half-reclining on the seats, some perched on the backs of the benches. They are waiting for the professor who lectures to them to-day. They come from all parts of the wide Austrian empire, stay about two months, are instructed free of expense, have regular practice in the wards, and are sent back home again, to attend, after a fashion, at the advent of the future subjects of his imperial majesty.

The mother, after the birth of the child, remains in the institution only nine days, in case all goes well, and makes her exit, leaving her babe behind her.

Would you like to know what becomes of these little innocents? Thereby hangs a lamentable story. The child, if strong, is sent at once out into the country to nurse: poor women taking five or six at a time for a mere pittance, and bringing them up as they best can. One-half die during the first year. A register is kept. The name of the mother and the date of her entry are entered there, and a piece of tape with the number of the birth on that year marked on it with indelible ink is sewed round the left wrist of the child. This is kept carefully on the wrist until the child is 6 years old, up to which period the mother may reclaim it. The name of the nurse is also registered, and, as she never lives more than a few miles distant, the mother can call and inquire after the infant. If the child is maltreated in any way, it is recalled and another nurse substituted.

I was witness to a scene where an infant, bearing on its arm the No. 2478, had been brought back because the nurse had beaten it, and the mother, a decently-clad woman, had come to see it. She mourned over it like another Rachel, refusing to be comforted, calling it her little angel, and many other endearing epithets, which I could hardly make out between her sobs and sighs, and her broken language.

Many of the young infants are too delicate to be sent at once into the country. They are transferred to the foundling hospital across the street, and such of the young mothers as are capable of nursing two children, are placed over there for three months, in order to take them under their charge. Admirable order is preserved in this institution, but it presents a miserable spectacle.

The selfish instincts of poor human nature would always lead the mother to foster more kindly her own offspring than the little stranger she was constrained at the same time to hold to her breast. I could often distinguish the wan, often sightless face of the one, from the plump, rosy cheek of the other, and would win a smile from the young, earnest face of the mother by saying, 'this is your own.' The number of children constantly connected with this institution is 20,000. Many die; few are reclaimed. At the ages of six or seven they are bound out to service, and, if boys, usually find their destination in the army.

But enough of such a subject. Let us pass on to Berlin, and see that distinguished pathologist, enlightened statesman, and amiable gentleman, whose name is familiar to you all. His genius is of so versatile a nature that often on the same day he will give a lecture on some obtruse point in pathology, and a flowing oration from his place in the chamber of representatives.

I had the pleasure of listening to the first two lectures of his course, which are called his railroad lectures. The room he occupies was built expressly for him; it is about fifty feet square. The floor and seats are on a level, and the tables so arranged in zigzag lines that a little railroad track, with turn-tables at the corners, is continuous round the room. On this track nine microscopes were mounted on wheels, and made to pass around the room by being pushed on from student to student, as each one examined the object displayed.

The Professor, of middle age, medium height, and prepossessing appearance, stood in one corner of the room, with a blackboard and towel hanging behind him. Five students were in another corner arranging the microscopes. Before these were put in motion, pathological preparations on dishes were passed around the tables, under the noses of the students. The Professor would describe one specimen, and set it agoing, then take up another. Thus four or five would be put in motion successively, and be making at the same time the circuit of the room. It was evident that the students had great difficulty to connect the descriptions they had heard with the right preparations. The same difficulty, too, must attend the use of the microscopes.

At the second lecture the main scenic effect consisted in the arrangement on a large table of a dozen wooden platters filled with pathological specimens. In one there was the skullcap and brain of a man who had died from a blow on the head which had produced suppuration between the membranes. The spleen and the liver on another, showing the commencement of metastatic abscesses. Here a portion of a pelvis showing the early stage of inflammation in the hip joint. On another plate were the lungs and heart. The great and small intestines were displayed on another, the latter ridded out and split open, and laid in longitudinal rows like the ruffles on a shirt. And again, we had other plates containing the bladder, and kidneys and diseased mesenteric glands.

The object of the second lecture was to show the effects of inflammation on the different tissues according to Virchow's peculiar theory.

As Schwann showed all living bodies to consist of a series of cells, and all the normal acts of life to consist of cell-actions, so Virchow reduces all morbid processes to merely morbid cell-action; the effused lymph or plasma itself which occurs in inflammation consisting of substances that form cells.

The preparations on the table were briefly described, with the bowels and the diseased mesenteric glands. These were made the subject of a lecture for three-quarters of an hour, in which he used freely and neatly the blackboard. The object of the lecturer was to contrast the pathological differences between exanthematous and abdominal typhus.

The lecturer was not in this place oratorical, and there was good taste in that. He held his head down somewhat, hesitated often, seemed much communing with himself, stood now with one hand in his trowsers pocket, now with two, varying the position occasionally by putting them in his coat pockets behind his back; but he was learned in his subject, and clear, and occasionally rose into animation. He is surely a man of distinction, and every way worthy of the high reputation he has achieved. I would that I had time here, which I have not, to give you his views more in detail.

Of the surgeons of Great Britain, with whose opinions in our common language you must needs be more familiar, it is not necessary that I should here say much, even were longer time allowed me. I shall confine myself, in this brief notice, to the two more novel points in surgical practice there, the value of which cannot as yet be considered settled: I allude to the use of acupressure after the manner of Sir James Y. Simpson, and the carbolic acid antiseptic treatment, which Prof. Lister, of Glasgow, now of Edinburgh, following some of the French surgeons, is most thoroughly investigating.

Acupressure is no new subject in this city I myself introduced its use some years ago into this clinic and in my service at the Pennsylvania Hospital, and its practical value has since been more fully observed by the present distinguished surgeons of that and other institutions in this city. Its object is, as you know, to secure the bleeding vessels without killing a piece of flesh with the ligature, and leaving that as a dead part in the wound, to provoke suppuration and endanger pyæmia. The practice of acupression, I observed, was growing into favor in many parts of the Continent, and even Prof. Syme, of Edinburgh, who has always opposed the method, pays a silent tribute to it, by substituting for the ligature the old-fashioned method of twisting the arteries, even vessels of the size of the radial and ulnar after the manner of Amussat. Professors Pirrie and Keith, of Aberdeen, have done most in perfecting this process, and find a wire loop, with a quarter twist of the wire on the needle, sufficient even for the larger arteries. It is impossible to say how far as yet we can extend beneficially the use of the method of acupressure. It is now used in a very simple way, to make pressure on the trunk of a vessel, in order to arrest the bleeding from some of its distant branches. And I think it quite possible it may yet be found reliable in the treatment of some forms of aneurism. Surely we must all admit that we owe a great debt to that distinguished obstetrician and good man, Prof. Simpson, for its introduction.

The antiseptic treatment, by the use in various ways of carbolic acid, is considered by the distinguished Prof. Syme as marking a new era in surgical treatment. But its value is by no manner of means generally established. Prof. Lister, the son in-law of Mr. Syme, is its greatest advocate. The theoretic grounds upon which the doctrine is erected, are these: That there are septic germs floating everywhere in the atmosphere, to be found even in the dust that accumulates on the end of the finger, and which, if they come in contact with the surface of a wound, are one of the chief causes that excite to suppuration. The carbolic acid kills these germs, so that if the surface of the wound is covered with this acid, diluted with water or oil, or mixed in a plaster, so as to completely exclude the air, there ought to be little or no suppuration. That there is great advantage to be obtained from the use of carbolic acid in this way, and in preventing suppuration after compound fractures, I have no manner of doubt, and Prof. Lister showed me many cases where, after serious operations, there had been no suppuration whatever, or but very little. Indeed, in a little work by the late Prof. John K. Mitchell, of Jefferson Medical College, you will find a sort of outline of these same doctrines in his cryptogamous theory of the production of disease from atmospheric germs. The cool and fine climate of Scotland, and the airiness of their hospital wards in general, must have much to do in the healing of wounds without suppuration. It is no uncommon thing either in this city, as many of us can attest. In Aberdeen Prof. Pirrie showed me cases where there had been similar good results without the use of carbolic acid, which he attributed to the use of acupression and their bracing climate. And among the cases he showed me were amputations of the breast, leg, and upper jaw.

